# Dispersal Characteristics Dependence on Mass Ratio for Explosively Driven Dry Powder Particle

**DOI:** 10.3390/ma16134537

**Published:** 2023-06-23

**Authors:** Binfeng Sun, Chunhua Bai, Caihui Zhao, Jianping Li, Xiaoliang Jia

**Affiliations:** 1 State Key Laboratory of Explosion Science and Technology, Beijing Institute of Technology, Beijing 100081, China; 2 Jinxi Industries Group Co., Ltd., Taiyuan 030027, China; 3 Liaoning Jinhua Electromechanical Co., Ltd., Huludao 125000, China

**Keywords:** explosive dispersal, dry powder, mass ratio, particle jet

## Abstract

An investigation on the dispersal characteristics of the cylindrically packed material of dry powder particles driven by explosive load is presented. By establishing a controllable experimental system under laboratory conditions and combining with near-field simulation, the particle dispersal process is described. Additionally, Kelvin–Helmholtz instability is observed during the process of jet deceleration dispersal. The characteristic parameters of radially propagated particles are explored under different mass ratio of particle-to-charge (*M*/*C*). Results indicate that, when the charge mass remains constant, an increase in *M*/*C* leads to a decrease in dispersed jet number, void radius and maximum velocity, wherein the maximum velocity correlates with calculations by the porous Gurney model. The case of the smaller *M*/*C* always has a higher outer-boundary radius and area expansion factor. Findings indicate that when particles detach from the jet upon reaching minimum acceleration and entering low-speed far-field stage from high-speed near-field stage, the outer-boundary radius is 30~36 times the initial particles’ body radius under different *M*/*C*. In addition, particle concentration distribution over time and distance is qualitatively analyzed by the grayscale image method. This research can be referential for improving the fire-extinguishing capacity of extinguishing bombs and the damage property of fuel air explosive (FAE).

## 1. Introduction

Explosive dispersal of the granular materials is extensively applied in industrial security and military engineering [1,2,3,4,5]. Dry powder fire-extinguishing bombs explode and disperse the dry powder particles to form stable aerosols, serving for fire control and suppression [6]. Fuel air explosive (FAE) brings about severe damage to the target by detonating the dispersed cloud driven by explosive load [7,8]. Among these application fields, it is of great significance to investigate the evolution law in the process of the particle dispersal, involving relevant parameters such as dispersal velocity and cloud area, etc. Such parameters can determine the fire-extinguishing capacity of the dry powder fire-extinguishing bomb, as well as the secondary initiation time, dropping position, and damage power of FAE.

In typical explosive dispersal, it is often a research challenge to obtain the detailed temporal visualization of the interface for high-pressure gas/particle contact and the particle/air contact, because these experiments are usually conducted in outdoor environments where the lighting and environment-related parameters are difficult to be controlled [9,10]. Zhang et al. [11] conducted a large-scale spay detonation using gasoline explosive load. Such an outdoor dispersal experiment allows only the outer-trajectories of the clouds to be observed, while the particles’ concentration distribution in the cloud region and the morphology of the inner-void are difficult to be accurately observed by optical shooting. To clearly obtain the dispersed cloud trajectory, small-scale dispersal experiments based on the laboratory environment have been carried out, mainly involving the use of the shock tubes or detonation tubes to generate shockwaves and drive particle dispersal [12,13,14]. The shockwave generated by shock tubes or detonation tubes belongs to weak shock load on the order of 10^−1^~10^0^ MPa, while the blast wave driven by explosive load is on the order of 10^1^ GPa. There is significant difference in load strength between the two [15]. Therefore, the dispersal experiments conducted using weak shock load are difficult to simulate the real particles’ dispersal driven by explosive loads in real situations.

The initial stage of explosive dispersal involves research problems such as the interaction between shockwaves and particles, detonation products and particles, as well as the interaction among the particles. However, this process occurs inside the particles’ body within the microsecond level, making it hard to be recorded with high-speed shooting. To better explore the evolution of particle dispersal driven by explosive load, it is necessary to conduct studies in a synchronous method by a combination of experimental and numerical simulation.

This paper focuses on the acquisition and research of the characteristic parameters in a dry powder material dispersal system driven by explosive load. A particle dispersal test system under controllable laboratory conditions is established, and the dispersal process of the annular dry powder material driven by a central detonator is experimentally investigated. By using a surface light source as the background light, the complete particle dispersal morphology, including inner- and outer-trajectories, as well as the grayscale distribution are captured more visibly compared to previous tests [3] carried out under natural light. To obtain the initial-stage particle dispersal process that cannot be measured experimentally, a numerical simulation method based on the smoothed particle hydrodynamics finite element (SPH–FEM) coupling algorithm is proposed, followed by a comparison of the results with the porous Gurney models. The characteristic parameters, including the number of jets and the maximum velocity in the dispersal process, are investigated under different mass ratio (the ratio of dry powder mass-to-charge mass (*M*/*C*)). Finally, the time-dependent laws of the characteristic parameters, including the dispersal radius, cloud area, the radius and the area of the explosive void, are explored. Particle concentration distribution is qualitatively analyzed by the grayscale image.

## 2. Materials and Methods

### 2.1. Dry Powder and Central Charge

**Figure 1:**
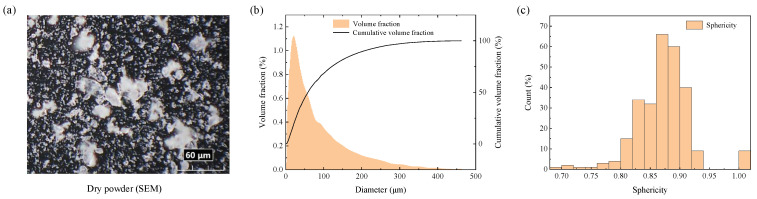
Dry powder material properties. (**a**) SEM image, (**b**) particle size (the median diameter D50 of the whole particles is 58.4 μm) and (**c**) sphericity distribution.

Dry powder extinguishant (75% NH_4_H_2_PO_4_, 15% (NH_4_)_2_SO_4_, 9% Mg_3_[Si_4_O_10_](OH)_2_, purchased from Zhengzhou Haichao fire agent Co., Ltd., Zhengzhou, China) is used for dispersal experiments, with a density of 1.8 g/cm^3^. The particle size and sphericity distribution, as well as the SEM (scanning electron microscope) image are shown in Figure 1. In the selection of central charge, factors including indoor test safety, space limitation, and the strength for fixing the device are comprehensively considered. The explosive equivalent should be minimized on the premise of ensuring the experimental effect. Accordingly, No. 8 electric detonator with a diameter of 7 mm is adopted as the central charge. It contains 1.074 g RDX, 30 mm behind the tail of the detonator.

### 2.2. Experimental Setup

**Figure 2:**
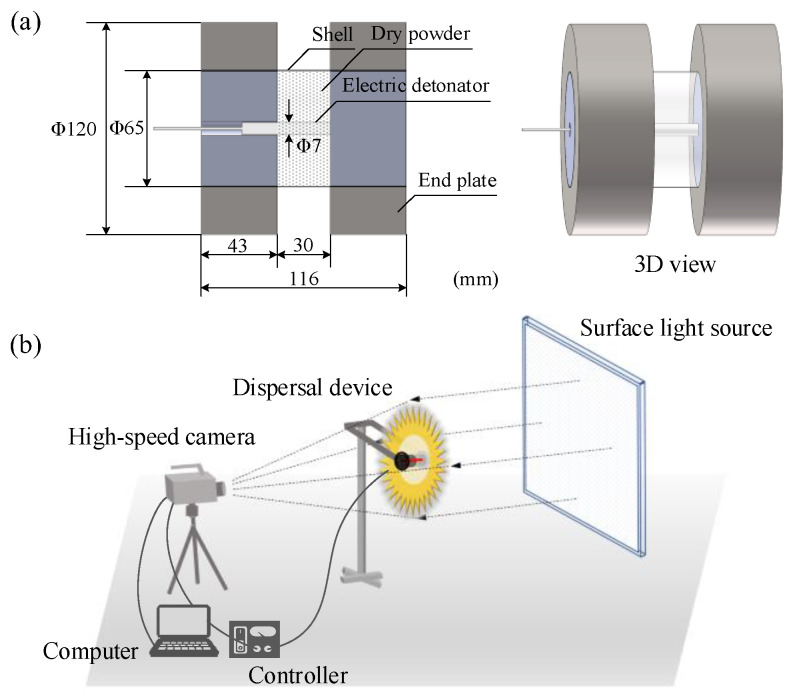
Diagram of (**a**) the dispersal device and (**b**) experimental setup.

Considering the experimental requirements for environment stability (no wind or natural light) and a large space, an enclosed darkroom with an inner-dimension of 20 m × 10 m × 5 m and safety protection measures is selected for the test. A cylindrical explosive dispersal device is designed with a central electric detonator surrounded by annular dry powder particles. To eliminate the impact of the shell debris on the particle dispersal, A4 paper is used for the packaging of the dry powder. Each end of the shell configures a plate with a diameter slightly larger than that of the particles’ body, so as to constrain the axial movement of the particles at the initial explosive stage while not affecting the optical shooting. The radial movement is research-oriented here. Specific dimension of the dispersal device is shown in Figure 2a. The height of the particles’ body is set to be 30 mm. To clearly observe the morphology of the dispersed particle cloud and capture the grayscale images, a uniform, flicker-free, and brightness-adjustable surface light source is selected as the backlight. The dimension of the surface light source is set to 2 m × 2 m, considering the particle dispersal radius.

FASTCAM NOVA S16 high-speed camera is used to record the dispersal process driven by explosive, with a frame rate of 10,000 fps and a resolution of 1024 × 1024. A controller is configured for the synchronous trigger of the high-speed camera and the detonator. An absolute pixel length scale is established according to the dimension of the surface light source.

### 2.3. Numerical Simulation Methodology

Explosively driven particle dispersal involves matters including large-scale displacement and the deformation of the particles’ body. As for the dispersed particles, separate finite element (FEM)-based method is not applicable due to its disadvantages of requiring complex computation and a reduced reliability when referring to mesh deformation. While for the SPH method, it employs the space-independent particle as the computational domain as a Lagrange particle algorithm [16,17]. Particle motion is used to describe the deformation of materials, which solves the problem of calculating large deformation in FEM. However, compared with FEM, the SPH method is less efficient because it requires information of other surrounding particles to calculate the physical quantity of a single particle. FEM method has advantages such as simple structural modeling and a mature dynamic response analysis technology. Hence, smoothed particle hydrodynamics (SPH) algorithm coupled with FEM is adopted for numerical simulation. Thereinto, the end plate used to constrain the axial movement of particles is simulated by FEM and defined as SHELL element. The paper shell that has little effect on the movement is ignored. For charge and dry powder with large deformation, SPH-based simulation is performed in ANSYS/LS-DYNA software. Thus, both computational efficiency and reliability of simulation results are reconciled. The coupling between SPH particles and FEM elements is achieved through automatic nodes-to-surface contact algorithm, where SPH particles are defined as slave bodies and FEM units are defined as main bodies. The physical information transfer among structures is realized through the coupling algorithm of SPH and FEM.

The material model chosen to describe the RDX charge is MAT_HIGH_EXPLOSIVE_BURN with a Jones–Wilkins–Lee (JWL) equation of state expressed as [18]:

(1) $$P_{c}=A\left(1-\frac{\omega }{R_{1}V}\right)e^{-R_{1}V}+B\left(1-\frac{\omega }{R_{2}V}\right)e^{-R_{2}V}+\frac{\omega E}{V}$$

where *P_c_* denotes the pressure of the detonation product, *V* is the relative specific volume, and *E* is the internal energy per unit volume of the charge. *A*, *B*, *R*_1_, *R*_2_ and *ω* denote the parameters of the JWL equation of state, as shown in Table 1 [19]. *ρ_c_* is the charge density and *D* is the detonation velocity.

Dry powder is described by the MAT_SOIL_AND_FOAM model [20], whose ideal plastic yield function can be expressed as:

(2) $$\phi =\frac{S_{ij}S_{ij}}{2}-(a_{0}+a_{1}P_{m}+a_{2}P_{m}{}^{2})$$

where *a*_0_, *a*_1_ and *a*_2_ are user-defined constants. *S_ij_* denotes the deviatoric stress component and *P_m_* (*m* = 1~10) denotes the pressure value at the corresponding points where volume strain occurs. The main calculation parameters are listed in Table 2, where *ρ*_0_ is the density, *G* denotes the shear modulus and *KUN* denotes the bulk modulus for unloading. *PC* and *VCR* denote the pressure cutoff for tensile fracture and volumetric crushing option, respectively. *EPS_m_* denotes the volume strain values.

**Figure 3:**
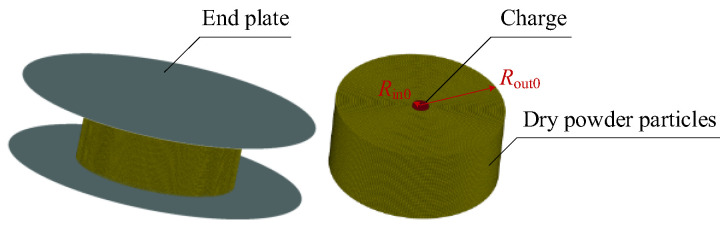
SPH–FEM coupling simulation model (*M*/*C* = 102.4).

Here, the deformation of the end plate is not considered in the simulation. In order to improve the calculation efficiency, MAT_RIGID model is used for description of the end plate, having a density of 7.8 g/cm^3^, Young’s modulus of 210 GPa and Poisson’s ratio of 0.3. The displacement and rotation in all directions are constrained. In the initial stage of the explosive dispersal, the shockwave generated by the explosion is much greater than the atmospheric pressure, thus ignoring the air resistance. The numerical simulation model (*M*/*C* = 102.4) of dry powder particles driven by charge is shown in Figure 3, where dry powder contains 787,140 SPH particles and charge contains 11,700 SPH particles. *R*_in0_ and *R*_out0_ denote the initial radius of the central charge and dry powder particles, respectively.

## 3. Results and Discussion

The dispersal evolution law of the dry powder particles driven by RDX charge under different mass ratio (ratio of the dry powder material mass to charge mass, *M*/*C*) was studied experimentally and numerically. *M*/*C* was changed by adjusting *R*_out0_, while the bulk density, height of the dry powder particles and the central charge-related parameters remained constant. Five operation conditions (*M*/*C* = 14.1, 28.8, 48.5, 73.0 and 102.4, respectively) were selected for subsequent analysis. Specific parameter setup is shown in Table 3.

### 3.1. Particle Dispersal Process and Morphology

Previous studies by Loiseau et al. [9] have revealed the spherical materials’ dispersal driven by a C-4 charge. The spherical materials’ dispersal is omni-directional. Only the morphology of the outer-layer particles’ cloud can be obtained while the inner-layer particles’ cloud is not visible due to being surrounded by outer-layer particles. In this study, the dry powder particles’ body in a cylindrical profile with a constrained axial movement can clearly exhibit the particles’ morphology of both the inner- and outer-trajectories in a radial direction. Moreover, the detachment process of the jet tip fragments at the later dispersal process is clearly observed, which is not realized in previous studies.

**Figure 4:**
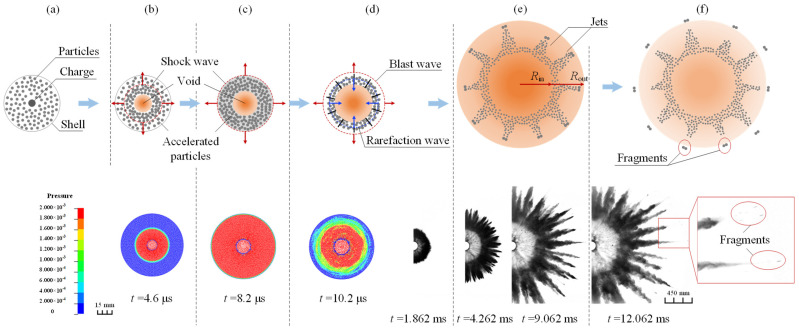
Dry powder particle dispersal process driven by cylindrical charge. Top (**a**~**f**) depict the diagram of the whole dispersal process. Bottom left presents the simulated process at the initial dispersal stage that is unavailable to be experimentally measured (*M*/*C* = 102.4). Bottom right displays the experimental images taken at the later dispersal stage (*M*/*C* = 102.4).

A diagram of the particle dispersal driven by explosive charge is depicted in Figure 4. The cross-section of the initial dispersal structure is shown in Figure 4a, appearing as a cylinder with central charge surrounded by dry powder particles. After the explosive detonation, the aroused shockwave propagates radially outward and compresses the porous particles to be compact. Simultaneously, the detonation product drives particle dispersal to form an inner-void (Figure 4b,c). The blast wave is then transmitted into the surrounding air, and the rarefaction wave travels inward through the compacted particles. The compacted particles expand under tension and then break into fragments (Figure 4d). A radial jet structure forms as the fragments propagate radially outward and the unconsolidated loose particles then fall off (Figure 4e,f). The velocity of the jet rapidly decreases and begins to slowly disperse as the consolidated fragments detach from the jet at a high speed. The lower part of Figure 4 presents the simulation (at the initial dispersal stage) and experimental images (at the later dispersal stage) corresponding to the different stages of the dispersal process under *M*/*C* = 102.4.

**Figure 5:**
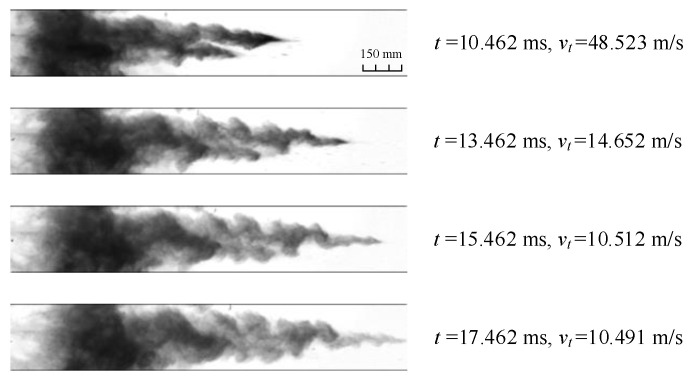
Kelvin–Helmholtz instability observed during the process of jet deceleration dispersal (*M*/*C* = 102.4 and *v_t_* denotes the real-time velocity).

During the deceleration dispersal stage of particles jet, Kelvin–Helmholtz instability [21] is observed, as shown in Figure 5. It is caused by the tangential velocity difference at the jet/air contact interface, exhibiting obvious vortex profile. The emergence of Kelvin–Helmholtz instability increases the mixing velocity and degree of the particles and air near the jet/air contact interface.

**Figure 6:**
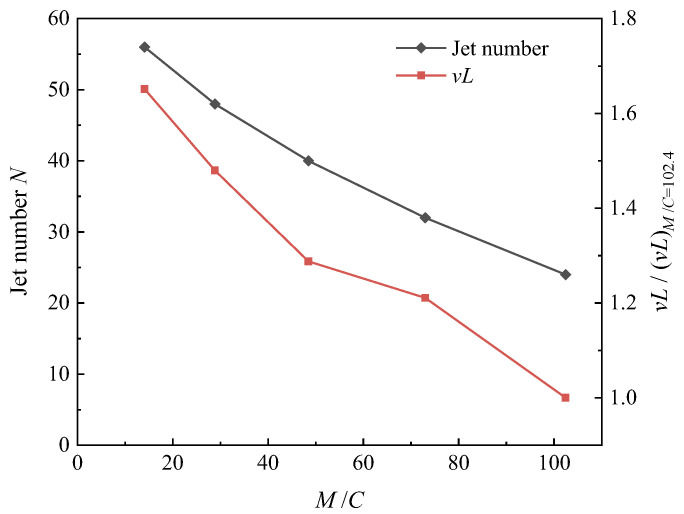
Dependence of normalized *vL* and the jet number *N* on mass ratio *M*/*C*.

Reynolds number *R_e_*, i.e., the ratio of inertial force to the frictional force in the particles system, is used to characterize the critical conditions for the jet formation. *R_e_* is expressed as [22]:

(3) $$R_{e}=\left(\rho vL\right)/\left(\gamma_{s}c_{s}d_{s}\right)$$

where *ρ*, *v* and *L* denote the loading density of the particles, maximum dispersal velocity and the thickness (*L* = *R*_ou0_ − *R*_in0_) of the particles’ body. *γ_s_*, *c_s_* and *d_s_* denote the mass density, sound velocity and the average diameter of the particles. Frost et al. [22] found that the jet number *N* increases with increasing Reynolds number. In this study, where the mass ratio *M*/*C* is taken as an independent variable, *v* and *L* are two variables that change accordingly. The experimental dependence of normalized product term *vL* and the jet number *N* on the mass ratio *M*/*C* is shown in Figure 6. It can be seen that *vL* is positively correlated with the jet flow number *N*, i.e., *R_e_* is positively correlated with the jet number *N*, which is consistent with Frost’s theory.

To obtain the trajectory radius and velocity of the dispersed particles, the experimental image is converted into a grayscale image, followed by a binarized operation with a self-defined grayscale threshold. The inner- and outer-boundary trajectories of the image are accordingly extracted. Then, a ray is drawn in each image from the charge center toward the direction of jet movement. The intersection points between the ray and the inner-/outer-boundary of the particles are defined as the inner-void radius *R*_in_/outer-radius *R*_out_ of the particles cloud, as marked in Figure 4e. The cloud area is extracted by the grayscale threshold on each image, and the inner-void area is calculated by *R*_in_. Following this, the characteristic parameters of the particle dispersal dependence on mass ratio *M*/*C* are presented, including maximum velocity, dispersal radius and area, the characteristics of the dispersed void, as well as the grayscale distribution of the dispersed particles.

### 3.2. Particle Dispersal Maximum Velocity

Previously, Gurney [23] proposed a model to predict the initial/maximum velocity of spherical and cylindrical homogeneous shells driven by high explosive charge. However, for the heterogeneous particle system in this study, there are pores among the particles. Under high explosive load, the compaction and deformation of the particle body can collapse and heat the pores, leading to a significant entropy dissipation of the explosive energy in the interstitial air and particles. Therefore, the standard Gurney model is no longer applicable for the valid prediction of the maximum velocity of granular material. Taking into account the porosity and bulk density effect, Milne [24] empirically modified the Gurney model, which is now expressed as:

(4) $$V_{\mathrm{Gurney}}\left(M/C\right)=\sqrt{2E}{\left(M/C+0.5\right)}^{-0.5}$$

(5) $$V\left(M/C,\rho_{0},\varphi \right)=V_{\mathrm{Gurney}\;}\left[\frac{M/C}{a\left(\rho_{0}\right)}\right]\times F\left(\varphi ,M/C\right)$$

(6) $$\alpha \left(\rho_{0}\right)=0.2\rho_{0}^{0.18}$$

(7) $$F\left(\varphi ,M/C\right)=1+\left(0.162e^{1.127\varphi }-0.5\right)\log_{10}\left(M/C\right)$$

where *M* is the mass of the powder, *C* is the mass of the charge, $\sqrt{2E}$ is the Gurney velocity coefficient (2.93 km/s for RDX [25,26]). *ρ*_0_ is material density and *φ* is the loading density (*φ* = *ρ*/*ρ*_0_ = 1.1/1.8 ≈ 0.6, where *ρ* denotes the bulk density).

**Figure 7:**
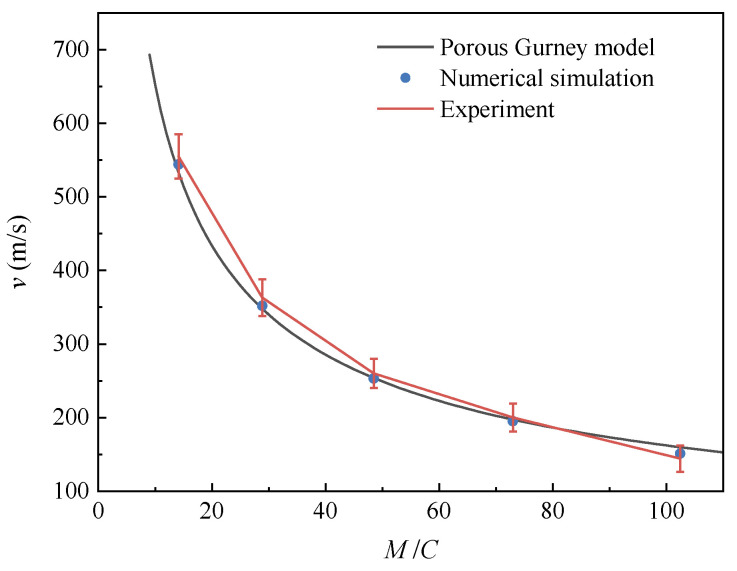
Dependence of maximum dispersal velocity on mass ratio *M*/*C*.

The maximum velocity of the particles under different *M*/*C* is given in Figure 7, including the results by experiments, numerical simulation and the porous Gurney model. It can be seen that there is good consistency among the three, verifying the reliability of the simulation model. In the early expansion stage of the explosive products, the process can be considered as isentropic adiabatic expansion because there is no heat exchange between the products and the particles due to the rapid action [27]. When *M*/*C* is increased by increasing the particle mass *M* while the charge mass *C* remains constant, the kinetic energy obtained by the whole particles system under different *M*/*C* is approximate. According to the kinetic energy theorem, the maximum velocity of the particles tends to decrease.

### 3.3. Particle Dispersal Radius and Area

**Figure 8:**
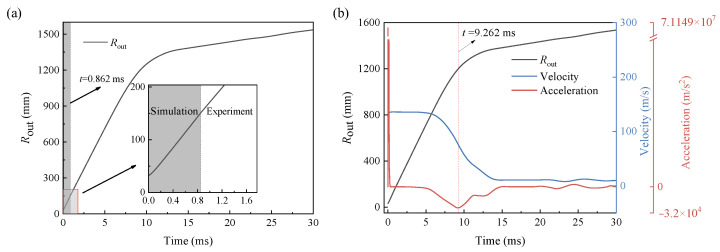
Time-dependent curves of (**a**) the particles’ outer-boundary radius, (**b**) the velocity, and the acceleration (*M*/*C* = 102.4).

To accurately obtain the variation of the dispersal radius and area over time, the starting time of the dispersal process need to be firstly determined. Although the high-speed camera shooting is controlled synchronously with the charging of the detonator, there are detonation time differences among the electric detonators, making it difficult to determine the starting time for every measurement. To address this problem, the simulated *R*_out_-time data under different *M*/*C* is referred. Taking the case of *M*/*C* = 102.4 as an example, the starting time when the first image is taken can be determined according to the simulated *R*_out_-time data. As shown in Figure 8a, the outer-boundary radius *R*_out_ extracted from the first image is 152.3 mm, then the corresponding time *t* = 0.862 ms is obtained by simulation. The simulated data are then concatenated between 0 and 0.862 ms with the experimental data after 0.862 ms, and a complete *R*_out_–time curve for describing the dispersal process is obtained. Accordingly, the time-dependent velocity and acceleration are given by performing first and second derivative operations on the *R*_out_–time curve, as shown in Figure 8b.

Previously, the process of explosive dispersal and cloud formation is divided into three stages [28]. As shown in Figure 8b, it is the acceleration at the near-field stage during 0~0.3 ms where the pressure from detonation products is greater than the air resistance; it is the uniform transition stage during 0.3~5 ms where the two are roughly equal; and after 5 ms, it is the deceleration at the far-field stage where the air resistance plays a dominant role. At the moment of minimum acceleration, the consolidated particle fragments detach from the cloud. Prior to this, the particle jet exhibits approximate high-speed ballistic movement; afterward, the movement velocity of the particles decreases rapidly and approaches zero. The moment (*t* = 9.262 ms) of minimum acceleration is marked in Figure 8b. According to the turning point of acceleration, the whole process can be re-divided into two stages: the near-field stage with high-speed motion and the far-field stage with low-speed motion.

**Figure 9:**
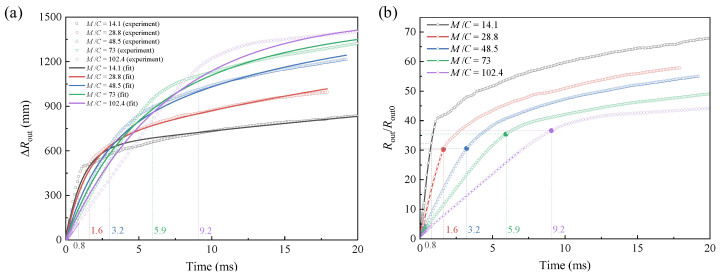
Time-dependent curves of (**a**) the outer-radius increment ∆*R*_out_ (experimental and fitting results, variance *R*^2^ = 0.997) and (**b**) radius expansion factor (experimental results) of the dispersed particles under different *M*/*C*. The moment when the minimum acceleration occurs is marked.

The time-dependent curves of the particles’ outer-boundary radius increment ∆*R*_out_ = *R*_out_ − ∆*R*_out0_ under different *M*/*C* are shown in Figure 9a. In the case of smaller *M*/*C*, ∆*R*_out_ increases faster at the initial stage, but the high-growth-rate duration is shorter than that of a larger *M*/*C*. Moreover, the initial ∆*R*_out_ is larger under a smaller *M*/*C*, but later, it is surpassed by the case with a larger *M*/*C*. This can be explained by the kinetic energy theorem. When the total kinetic energy obtained by the system remains constant, the particle body with a smaller mass can obtain a higher velocity at the initially explosive stage. However, the particles’ body is thinner, leading to the unconsolidated particles detaching earlier from the consolidated fragments. The loose particles have a smaller density and are subject to greater air resistance. Therefore, the particles’ body with smaller mass will ultimately achieve a smaller radius and displacement. The opposite accounts for particles’ body with a larger mass.

As a result, to determine the outer-boundary radius ∆*R*_out_, not only the initial velocity but also the duration of high-speed movement should be comprehensively considered. In order to quantitatively reveal the dependence of ∆*R*_out_ and mass ratio *M*/*C* on time, the experimental results are fitted, as shown in Figure 9a. The fitting expression (units: g-mm-ms) is given as follows:

(8) $$\Delta R_{\mathrm{out}}=1500-620\exp\left\{-t/\left[0.334e^{\left(M/C\right)/35}\right]\right\}-880\exp\left\{-t/\left[177e^{-\left(M/C\right)/14}\right]\right\}$$

The radius expansion factor, defined as *R*_out_/*R*_out0_, is time-dependent under different mass ratio *M*/*C*, as shown in Figure 9b. It shows a trend of rapid growth in the initial stage and gentle growth in the later stage. Under a smaller *M*/*C*, *R*_out_/*R*_out0_ increases faster at the initial stage but does not last long. With the increase in *M*/*C*, *R*_out_/*R*_out0_ has a smaller initial growth rate but lasts longer. Unlike the ∆*R*_out_–time curves, the *R*_out_/*R*_out0_–time curves always have a larger *R*_out_/*R*_out0_ at low *M*/*C* than at high *M*/*C*. Moreover, the particles under a smaller *M*/*C* possess a larger radius expansion factor than that of a larger *M*/*C* at the same moment. Provided that the particles’ body mass remains constant, a larger expansion factor, i.e., a larger outer-boundary dispersal distance, can be obtained by increasing the charge mass within a certain range. When the minimum acceleration is reached, *R*_out_ is 30~36 times that of *R*_out0_ as is marked in the vertical coordinate in Figure 9b.

**Figure 10:**
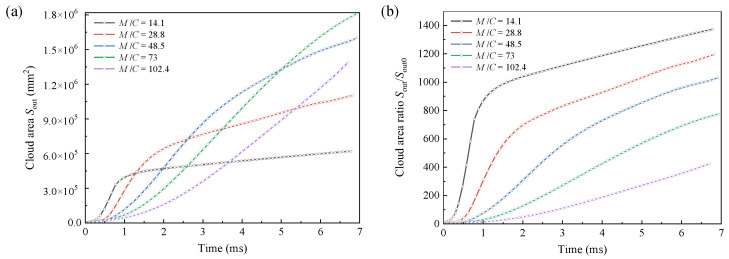
Time-dependent curves of the (**a**) cloud area and (**b**) area expansion factor.

Particle dispersal radius determines the dispersal distance. However, due to the structural morphology of particle jets, it is necessary to study the variation of cloud area to obtain the dispersal coverage range. The initial area of the particles’ body is defined as *S*_out0_, and the area *S*_out_ formed after dispersal can be obtained by *S*_out_ = *S*_out0_ + ∆*S*_out_ (∆*S*_out_ denotes the area increment). The time-dependent curves of the dispersal cloud area under different *M*/*C* are given in Figure 10a. The expansion factor of the cloud area, defined as *S*_out_/*S*_out0_, is time-dependent under different mass ratio *M*/*C*, as shown in Figure 10b. *S*_out_ and *S*_out_/*S*_out0_ exhibit a similar trend to that of the particles’ outer-boundary radius increment ∆*R*_out_ and radius expansion factor *R*_out_/*R*_out0_, respectively. Provided that the particles’ body mass remain constant, a larger expansion factor, i.e., a larger cloud coverage area, can be obtained by increasing the charge mass within a certain range.

### 3.4. Characteristics of the Dispersed Void

Previous studies [29,30] are unable to clearly obtain the internal explosive void formed by the particle dispersal. Through the experimental method here, a circular explosion void was clearly observed as demonstrated in Figure 4, which is defined as the region within the interface of the cloud, with a radius of *R*_in_.

**Figure 11:**
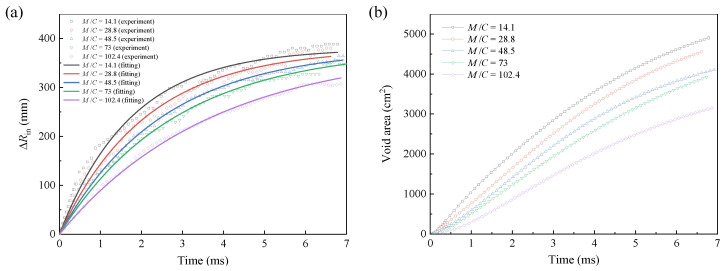
Time-dependent curves of (**a**) the void radius increment ∆*R*_in_ (experimental and fitting results, variance *R*^2^ = 0.991) and (**b**) the void area (experimental results) under different *M*/*C*.

The initial radius *R*_in0_ of the void is equal to the radius of the central charge, and the real-time radius *R*_in_ can be obtained by *R*_in_ = *R*_in0_ + ∆*R*_in_ (∆*R*_in_ denotes the radius increment). The time-dependent curves of ∆*R*_in_ and void area are shown in Figure 11. The results indicate that the variation of ∆*R*_in_ over time appears smooth without a significant turning point. The variation of void area over time tends to be more linear. As the pressure of the detonation products in the void gradually decreases, the resistance gradually dominates. Accordingly, the particles disperse slower, exhibiting a decreased slope of ∆*R*_in_–time curves. The void is generated by the expansion of the detonation products to promote particles dispersal. Under a larger *M*/*C*, i.e., a larger particle mass *M*, the resistance is larger, leading to a slower expansion of the detonation products. Accordingly, the generated void has a smaller radius.

The fitted results of the time-dependent ∆*R*_in_ under different *M*/*C* are shown in Figure 11a, and the expression is given as follows:

(9) $$\Delta R_{in}=380\left\{1-\exp\left[-t/\left(1.44856+0.02127\times \left(M/C\right)\right)\right]\right\}$$

### 3.5. Particle Dispersal Grayscale Distribution

**Figure 12:**
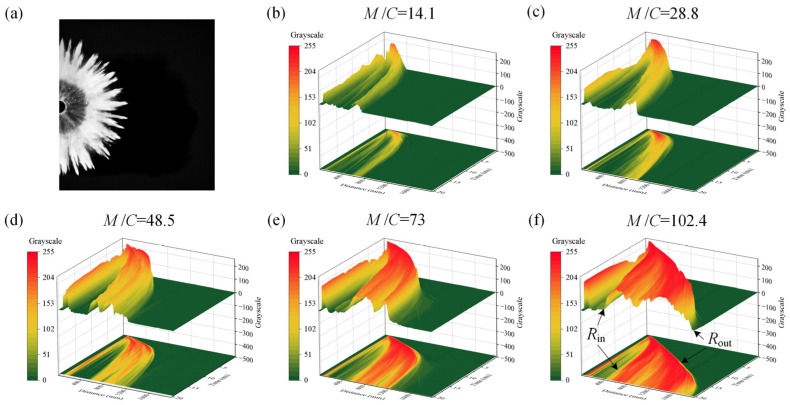
Particles’ grayscale extraction method and distribution. (**a**) Grayscale extraction. (**b**–**f**): Grayscale distribution of the dispersed particles under different *M*/*C*.

Particles’ concentration distribution can be qualitatively analyzed by the grayscale image. The grayscale image is obtained by subtracting the initial background image from the cloud image, as shown in Figure 12a, with a grayscale value between 0 and 255. The black pixels with a grayscale value of 0 represent the background, and the pixels with grayscale values between 1 and 255 represent the particles. The larger the grayscale value, the higher the concentration. Figure 12b–f depict the particles’ grayscale distribution over time and distance under different *M*/*C*. Compared with research conducted by Gao et al. [31], where an ultrasonic–electric hybrid detection method was adopted for partial-area particle concentration acquisition, here, the overall grayscale distribution in the cloud area can be obtained to analyze the overall concentration distribution law. The peak near the coordinate origin of distance axis is due to the frictional force between the particles and the end plate, causing a small portion of the particles to remain near the end plate. The inner-boundary radius *R*_in_ and outer-boundary radius *R*_out_ of the main part of the cloud are marked in Figure 12f. Over time, the cloud width (*R*_in_ − *R*_out_) increases and the concentration decreases. Radial bimodal fluctuation is presented in the main part of the cloud from the grayscale value variation over distance. Under a larger *M*/*C*, the cloud width and concentration are larger.

## 4. Conclusions

A controllable explosively driven particles dispersal system has been established under laboratory conditions for investigating the dispersal process. By conducting dispersal experiments on cylindrical particles’ body with a charge placed in the center, both the inner- and outer-dispersal trajectories in radial direction were clearly obtained. Through the combination of experimental and numerical simulation, the characteristic parameters evolution of dry powder particles dispersal under different mass ratio *M*/*C* was studied. Additionally, the particle concentration distribution over time and distance was qualitatively analyzed under different *M*/*C* by the grayscale image.

The research results indicate that when the charge mass remains constant, the jet number decreases as the mass ratio increases. Additionally, the outer-boundary radius and area of the cloud show a tendency of rapid growth in the early stage and gentle growth in the later stage. Moreover, the initial outer-radius and area of particles under low mass ratio conditions are larger, but later, they are surpassed by particles under high mass ratio conditions. The expansion factors of the outer-boundary radius and area are always higher under the small mass ratio in the whole process. When the outer-boundary reaches the minimum acceleration, the radius of the outer-boundary is 30~36 times the initial radius of the particle body. From this moment on, the particles move from a high-speed near-field stage to low-speed far-field stage. In addition, the radius of the void decreases with increasing mass ratio.

The proposed experimental system is not only applicable to the research of explosive dispersal for cylindrically packed granular materials, but can also be extended to the research of liquid or solid–liquid mixed materials with other shapes. It can be referential for improving the fire-extinguishing capacity of the extinguishing bombs and the damage property of FAE, as well as the performance prediction of the dispersal materials. SPH or an SPH-coupled simulation method can also be extended to other research fields involving thr prediction of non-military explosive characteristics such as mines, tunnel and structural demolition.

## Figures and Tables

**Table 1:** Detonation properties of the RDX charge and JWL equation-of-state parameters.

*ρ_c_* (g/cm^3^)	*D* (m/s)	*P_c_* (GPa)	*A* (GPa)	*B* (GPa)	*R*_1_	*R*_2_	*ω*	*E* (MJ/kg)
1.436	7500	17.5	100.3	22.2	6.028	1.8519	0.48	9

**Table 2:** Dry powder material parameters.

*ρ*_0_ (g/cm^3^)	*G* (GPa)	*KUN* (GPa)	*a*_0_	*a*_1_	*a*_2_	*PC*	*VCR*		
1.8	1.601 × 10^−2^	13,280	0.0033	1.31 × 10^−7^	0.1232	0	0		
*EPS*_1_	*EPS*_2_	*EPS*_3_	*EPS*_4_	*EPS*_5_	*EPS*_6_	*EPS*_7_	*EPS*_8_	*EPS*_9_	*EPS*_10_
0	0.05	0.09	0.11	0.15	0.19	0.21	0.22	0.25	0.3
*P*_1_ (GPa)	*P*_2_ (GPa)	*P*_3_ (GPa)	*P*_4_ (GPa)	*P*_5_ (GPa)	*P*_6_ (GPa)	*P*_7_ (GPa)	*P*_8_ (GPa)	*P*_9_ (GPa)	*P*_10_ (GPa)
0	3.42	4.53	6.76	12.7	20.8	27.1	39.2	56.6	123

**Table 3:** Parameters setup for the experiment and simulation.

No.	Charge Mass *C* (g)	Dry Power Mass *M* (g)	Mass Ratio (*M*/*C*)	Inner-Radius *R*_in0_ (mm)	Outer-Radius *R*_out0_ (mm)
1	1.074	15.17	14.1	3.5	12.5
2	1.074	30.98	28.8	3.5	17.5
3	1.074	52.05	48.5	3.5	22.5
4	1.074	78.39	73.0	3.5	27.5
5	1.074	110.00	102.4	3.5	32.5

## Data Availability

Not applicable.
